# Re-initiation of bottom water formation in the East Sea (Japan Sea) in a warming world

**DOI:** 10.1038/s41598-018-19952-4

**Published:** 2018-01-25

**Authors:** Seung-Tae Yoon, Kyung-Il Chang, SungHyun Nam, TaeKeun Rho, Dong-Jin Kang, Tongsup Lee, Kyung-Ae Park, Vyacheslav Lobanov, Dmitry Kaplunenko, Pavel Tishchenko, Kyung-Ryul Kim

**Affiliations:** 1 Seoul National University, Seoul, Republic of Korea; 2 Geosystem Research Corporation, Gunpo, Republic of Korea; 3 Korea Institute of Ocean Science and Technology, Ansan, Republic of Korea; 4 Pusan National University, Busan, Republic of Korea; 5 Far Eastern Branch, Russian Academy of Science, Vladivostok, Russia; 6 Gwangju Institute of Science and Technology, Gwangju, Republic of Korea

## Abstract

The East Sea (Japan Sea), a small marginal sea in the northwestern Pacific, is ventilated deeply down to the bottom and sensitive to changing surface conditions. Addressing the response of this marginal sea to the hydrological cycle and atmospheric forcing would be helpful for better understanding present and future environmental changes in oceans at the global and regional scales. Here, we present an analysis of observations revealing a slowdown of the long-term deepening in water boundaries associated with changes of water formation rate. Our results indicate that bottom (central) water formation has been enhanced (reduced) with more (less) oxygen supply to the bottom (central) layer since the 2000s. This paper presents a new projection that allows a three-layered deep structure, which retains bottom water, at least until 2040, contrasting previous results. This projection considers recent increase of slope convections mainly due to the salt supply via air-sea freshwater exchange and sea ice formation and decrease of open-ocean convections evidenced by reduced mixed layer depth in the northern East Sea, resulting in more bottom water and less central water formations. Such vigorous changes in water formation and ventilation provide certain implications on future climate changes.

## Introduction

**Figure 1:**
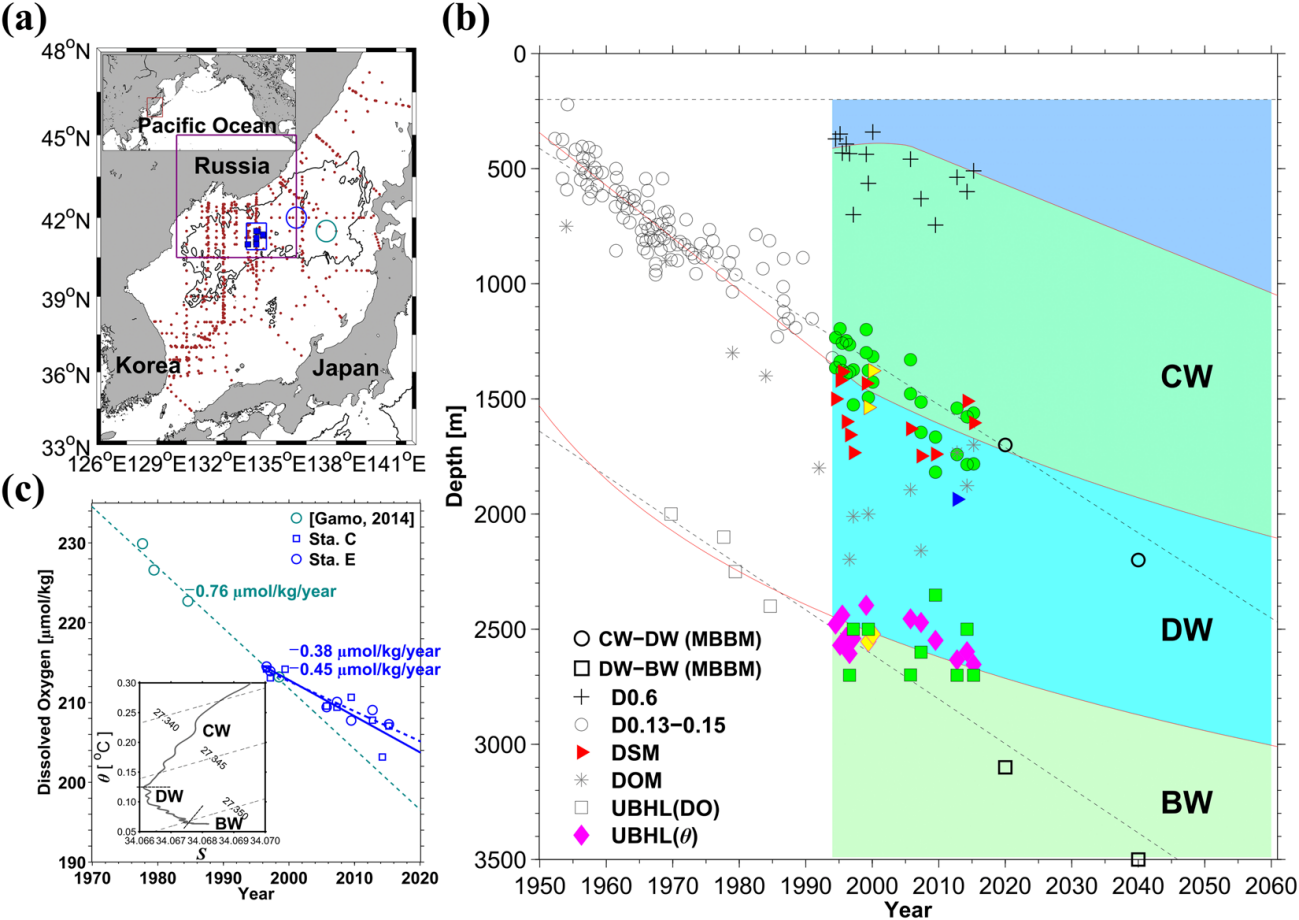
Deep structural changes in the East Sea (Japan Sea). (**a**) Geographic locations of the East Sea (ES) (brown box in the upper-left panel) and stations where the hydrographic data were collected through research cruises (brown dots). The Japan Basin (JB) is deeper than 3000 m (contour shown in thick black line), and the data collected in central JB or Station C (centred around 134°E, 41.3°N, blue squares) are mainly used here. Northern ES, where the surface air-sea fluxes are averaged (see texts for details) and other stations in eastern JB are marked by a purple box, and blue and green circles, respectively. (**b**) Time series of the boundaries between Central Water (CW) and Deep Water (DW) defined by 0.13–0.15 °C isotherms (open^2^ and green filled circles) or deep salinity minimum (DSM, red triangles), between DW and Bottom Water (BW) or upper limit of benthic homogeneous layer (UBHL, derived from potential temperature: magenta diamonds or dissolved oxygen: open^8,45^ and green filled squares), and depth of dissolved oxygen minimum (DOM, grey asterisk) for 67 years from 1950 to 2016^8,18^. Isotherms of 0.6 °C used for the upper boundary of CW are shown with crosses. Data collected in 1999 (June), 2000, and 2001 using different Conductivity-Temperature-Depth (CTD) instruments are shown with yellow triangles and diamonds (see Table 1). The DSM observed in 2012 is denoted with a blue triangle (see text). Three black dashed lines denote the upper boundary of CW fixed at 200 m, and linear fits to the observed CW–DW and DW–BW boundaries (from top to bottom), previously reported using data from 1950 to 1996^2,8,45^ (not shaded). Red solid lines are new fits to the up-to-dated boundaries. (**c**) Time series of BW dissolved oxygen (DO) observed from 1977 to 2015 in eastern JB (green open circles in Fig. 1a)^20^, Station C (blue squares in Fig. 1a), and Station E (blue circles in Fig. 1a). A green dashed line indicates a linear trend of declining BW DO in the eastern JB between 1970 and 2020, derived using data collected from 1977 to 1999. The blue dashed (solid) line shows for the same linear trend between 1996 and 2020 but with data collected at Station E (Station C) from 1996 to 2015. A potential temperature-salinity diagram for data collected at Station C in June 1999 is shown in the bottom-left inset. Figures were generated using MATLAB R2016a (http://www.mathworks.com).

The East Sea (ES) is a small marginal sea in the northwestern Pacific enclosed by countries such as Korea, Russia, and Japan (Fig. 1a). Its total area and average water depth are approximately 10^6^ km^2^ and 1,700 m, respectively. This deep marginal sea, connected to the Pacific through narrow and relatively shallow straits, has the highest deep-water dissolved oxygen (DO) in the Pacific because it is well ventilated through diverse processes such as brine rejection, convection, and subduction^1–5^. Using highly precise measurements, very cold and oxygen-rich deep-water masses in the ES, formerly called Proper Water^6^, have been subdivided into Central Water (CW), Deep Water (DW), and Bottom Water (BW)^4,5^. The deep salinity minimum (DSM) lies at a depth of around 1500 m, and defines the lower (upper) boundary of CW (DW)^4,5^. The DSM is shown in the potential temperature-salinity (*θ*-*S*) diagram in Fig. 1c. With rare *S* measurements of sufficiently high vertical resolution, isotherms of 0.13–0.15 °C were previously used to determine the boundary between CW and DW^7^. The upper boundary of CW was set to a 0.6 °C isotherm depth^4^. A benthic homogeneous layer (BHL) with a nearly constant *θ* or DO characterizes BW^4,8^ (e.g., *θ*-*S* diagram in Fig. 1c). A layer of DO minimum (DOM) is found between DSM and the upper boundary of BHL (UBHL), within the DW regime, presumably due to its relatively “old” age. The DOM structure indicates that formations of both CW (via open-ocean deep convection) and BW (via brine rejection and slope convection)^1,2,4,5^ are more active than that of DW.

Structures of deep-water masses indicate that ventilation in the ES has been maintained with significant changes since the 1950s^1,2,4,7–10^. Due to its rapid overturn timescale (<100 years)^1,11^, compared to that in the global ocean, and strong sensitivity to the formation of water masses^1,4^ well isolated from all subsurface waters in the Pacific, changes in the ventilation system of the ES would provide important implications on how world marginal seas and global ocean respond to future changes in climate. Results of the moving boundary five-box model applied to the ES suggested a shift in ventilation from the bottom to central/deep layers, resembling the weakening of deep ventilation in the global ocean^2,7^. Such shifts provide a clue to future changes in the global ocean^1,12,13^. Previously, BW was predicted to completely disappear by 2040 as CW replaces BW^7^. These projections were documented 13 years ago, and are re-visited here with hydrographic data collected between 1993 and 2016 (19 cruises in total). Temporal changes in the ventilation system are demonstrated with the structures of deep-water masses, and their association with winter ocean–atmosphere conditions in the northern ES is discussed. Then, future ventilation is newly projected based on a 1-D advection-diffusion model.

## Results

### Observations of deep structural changes

Deep structural changes are clearly observed at a group of stations (referred to as Station C), located in the central Japan Basin (JB), which is the deepest part of the ES (Fig. 1a). At Station C, the linear trend of the deepening of 0.13–0.15 °C isotherms as a proxy of the DSM^7^ from 1950 to the mid-1990s is approximately 19 m year^−1^ (open circles in Fig. 1b)^7^ and it has not significantly changed after the mid-1990s (green filled circles in Fig. 1b). However, the linear deepening trend of actual DSM is less than 7 (±11) m year^−1^ (though insignificant statistically at the 95% confidence level) after the mid-1990s (red triangles in Fig. 1b) when salinity data of high vertical resolution to define the DSM became available. The *S*-based DSM cannot be estimated for period before 1993 as the salinity data, if any, were collected with discrete water samples only. Therefore, it is not assured whether or not the deepening rate of the CW–DW boundary has been slowed down in 2000s as compared to the rate before 2000s, solely by the *S*-based DSM.

The DO-based UBHL has continuously deepened since 1950, but its deepening rate has significantly slowed down in the recent years (open and green filled squares in Fig. 1b). The linear deepening rate of the DO-based UBHL was 27 m year^−1^ (open squares in Fig. 1b) between 1969 and 1994. The deepening rate, however, reached close to zero between 1994 and 2015 (green filled squares in Fig. 1b). The *θ*-based UBHL (magenta diamonds in Fig. 1b) during the same period is 4 (±6) m year^−1^, which is insignificant at the 95% confidence level. Hence, the deepening rate of UBHL since 1950s has been significantly decreased after 1995 in terms of the DO-based estimate, and the *θ*-based estimate supports the recent slowdown of the UBHL deepening.

Based on these observations, two different fits were applied to represent trends of the deepening isotherms and/or DSM; linear fitting from 1950 to 1998 and logarithmic fitting from 1999 to 2015 extending up to 2060 (red lines in Fig. 1b). Regarding the UBHL based on both *θ* and DO profiles, only one logarithmic fit was applied for the entire period (1950–2060) (red lines in Fig. 1b). The fitting functions and periods were selected to optimize the results of the 1-D advection-diffusion model (see the next section for details).

The fitting lines might be considered sufficiently sensitive to contain significant decadal-scale variations of deep structural changes. The decadal variability in deep-water properties of the ES has previously been reported^4,14–17^ and changes of the Tsushima Warm Current^15^ and cold-air outbreaks related with the Arctic Oscillation^16^ were suggested as potential drivers underlying the variability. Watanabe *et al*. suggested a possible connection between decadal changes of nutrients in the ES deep-water masses and those in the North Pacific Intermediate Water^14^. It should be noted that the decadal variability in the previous studies is superimposed on the linear increasing trend for *θ* and decreasing trend for DO but without any change in their trend lines. This is plausible since data used for those studies are all acquired before 2005, when the linear trends dominated and slowdown of their changes cannot be fully accounted for in their studies. Our irregularly obtained data show significant scattering due to interannual as well as decadal variations, although a hint for the decadal variability is recognized. Considering the limitation of the observed data, we focused on changes in the longer-term trends (multi-decadal timescale) with significant changes in deep-water properties since the 1950s. The results are more related with previous attempts to project future changes, e.g., concerning an anoxic condition by 2200^18^, disappearance of BW by 2040^7^, and shift in the ventilation mode from more BW to more CW formations^7,19,20^.

The CW–DW boundary is defined as the DSM and fitting to the observed data is optimized against the 1-D model results (see Section 2.3 for details). From 1965 to 2020, the CW–DW boundary was deeper than the linear fit of the boundary projected by the moving boundary five-box model^7^. This indicates more accelerated and decelerated CW–DW boundary deepening before and after the 1990s, respectively, than that projected with constant water formation (red solid vs. black dashed lines in Fig. 1b). Such a slowdown in the CW–DW boundary deepening rate after the mid-1990s is more consistent with the DSM variations than the continuously deepening 0.13–0.15 °C isotherm depths. Thus, the fit optimized to the 1-D model supports that the DSM definition in determining the CW–DW boundary would be appropriate for resolving deep structural changes in the ES at least in recent decades. Such changes in the deepening rate of the CW–DW boundary yielded the DSM depth shallower by ~270 m in 2040 than that in the previous projection, e.g., new and old projections yield 1930 and 2200 m, respectively. The Root-Mean-Squared (RMS) differences of the old (linear fit)^7^ and new projections against the observations (DSM) are 125 and 114 m, respectively (dashed line vs red triangles for old projection, and solid line vs red triangles for new projection, Fig. 1b).

More interestingly, the UBHL, also optimized for the 1-D model, is markedly shallower than the previous projection, e.g., 2700 m vs. 3100 m for 2020 (Fig. 1b), showing that BW will not disappear by 2040 as previously suggested^7^. The RMS differences of the old (linear fit)^7^ and new projections against the observations (UBHL) are 155 and 76 m, respectively. Consistent variations of the DSM and UBHL were found using data collected at other stations in the JB (not shown).

The UBHL deepening is accompanied by declining BW DO in the JB, which also slowed down in the 2000s (Fig. 1c). The linear rate of the decreasing BW DO was estimated to be −0.76 μmol kg^−1^ year^−1^ during 1977–1999 for eastern JB^20^ (green circles in Fig. 1a and c and green dashed line in Fig. 1c), whereas the trend was significantly relieved to −0.45 μmol kg^−1^ year^−1^ and −0.38 μmol kg^−1^ year^−1^ during 1996–2015 based on DO observations at Station C (blue squares in Fig. 1a and c and solid line in Fig. 1c) and Station E (blue circles in Fig. 1a and c and blue dashed line in Fig. 1c), respectively. The slowdown of the decline in BW DO in the 2000s is consistent with the slowdown of the UBHL deepening, supporting the formation of more BW than that in the last decade.

### 1-D advection-diffusion model

A steady state 1-D advection-diffusion model^21^ was used to examine deep-water structure with the simple balance between vertical advection and diffusion/mixing. Assuming that *θ*, *S*, and DO (*DO*) of the ES is horizontally homogeneous below 500 m, and the system is in a quasi-steady state mode, the 1-D advection-diffusion model can be applied to account for changes in the deep-water structure from 1950 to 2016.

1 $$0=K(\frac{{d}^{2}q}{d{z}^{2}})+w(\frac{dq}{dz})+J$$

**Figure 2:**
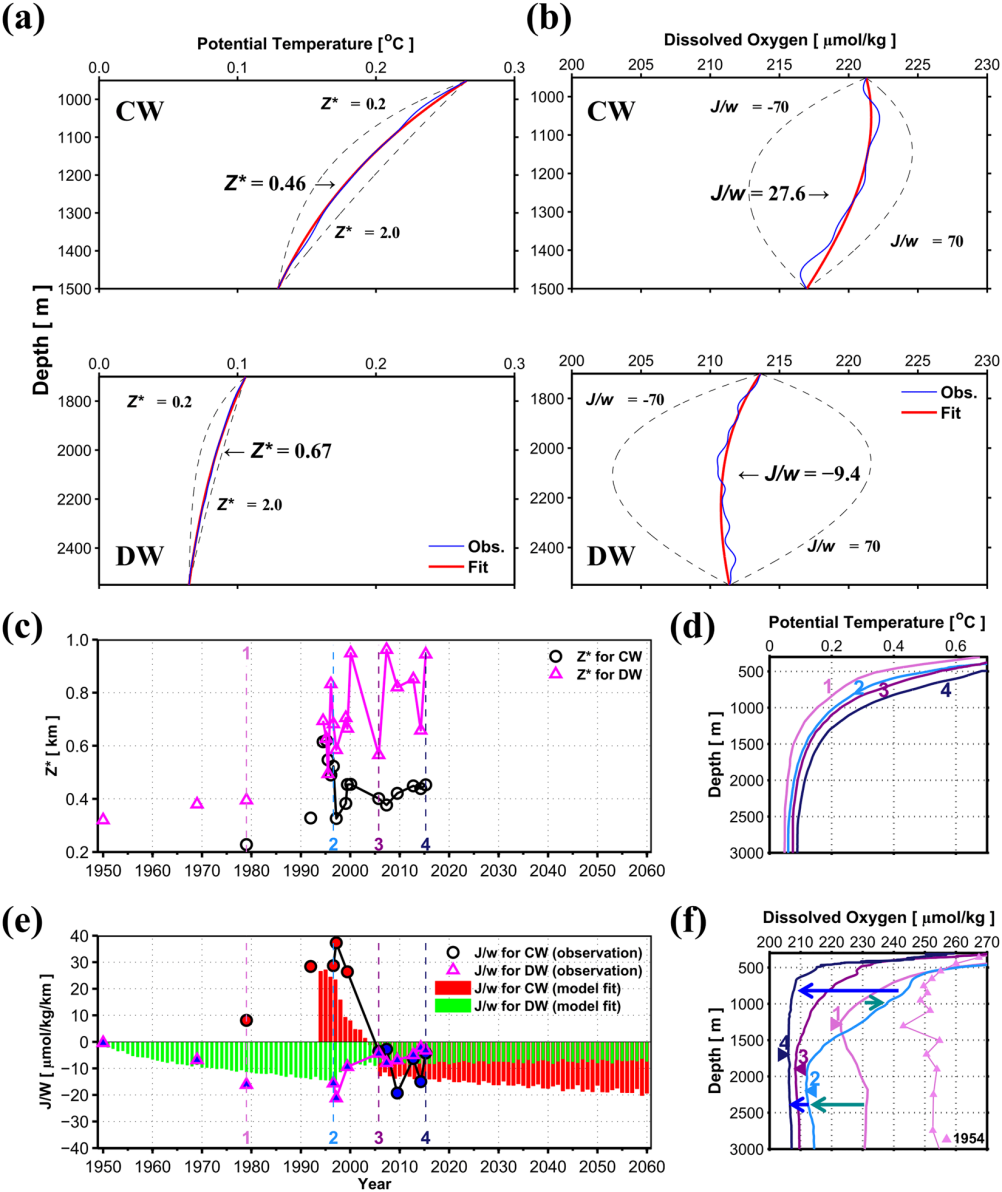
Results of one-dimensional advection-diffusion model. (**a,b**) Example of vertical profiles of potential temperature and dissolved oxygen from both observations (blue) and 1-D advection-diffusion model fits (red) to best determine Z* (*K/w*) and *J/w* for CW and DW. Range of Z* (*J/w*) to be tested is 0.2 to 2.0 km (−70 to 70 μmal/kg/km) (**c**) Time series of Z* at Station C. Z* values for CW and DW are shown with magenta triangles and black circles, respectively. (**d**) Vertical profiles of potential temperature for four periods marked with vertical dashed lines in (**c** and **e)**. (**e**) Time series of *J/w* at Station C. *J/w* values for CW and DW are shown with magenta triangles and black circles, respectively, filled with red (positive) and blue (negative) colours. *J/w* for CW and DW, independently estimated from equation (3) (*Model J/w*), is shown with red and green vertical bars, respectively. (**f**) Same as (**d**) but for DO. A vertical DO profile for 1954^1^ is also represented by violet triangles and the line. Changes in vertical DO profile from 1954 to 1979 (no. 1) to 1996 (no. 2) and from 1996 (no. 2) to 2005 (no. 3) to 2015 (no. 4) denote “a clue” (green arrows) and “another clue” (blue arrows) to future changes in the global ocean. Figures were plotted using MATLAB R2016a (http://www.mathworks.com).

Here, *q* indicates the variables *θ*, *S*, and *DO*; *K*, *w*, *J*, and *z* are diffusion or mixing coefficient, vertical velocity, source (*J* = 0 for *θ* and *S*), and the vertical axis, respectively. Ratios between *K* and *w* (Z* = *K*/*w*) and between *J* and *w* (*J*/*w*) are calculated for CW and DW from the observed *θ* and *DO* profiles independently. These ratios represent the relative importance of downward heat diffusion/mixing (warming) vs. upward advection or upwelling (cooling), and that of oxygen supplied into the corresponding water mass directly from surface source water (increasing oxygen) vs. upward advection of oxygen plus net biological consumption (decreasing oxygen). The upwelling (*w* > 0) is inferred from the curvature of *θ* profile (Fig. 2a,d), and the sign of *J*/*w* or *J* indicates whether the oxygen is convectively (or for any other mechanism) added. Vertical profiles of *θ* and DO in each layer obtained using equation (1) for Z* ranging from 0.2 to 2.0 km and *J/w* ranging from −70 to 70 μmol kg^−1^ km^−1^ are fitted to the observed profiles (blue vs. red solid lines in Fig. 2a and b).

The Z* values for CW and DW obtained from the *θ* profiles (Fig. 2d) generally increased with time from 1950 to 2015 (Fig. 2c), indicating an enhanced diffusion/mixing and/or weakened upwelling. However, they remained below 1.0 km, and they were always higher for DW than for CW. Such high Z* values for DW in the last decade is more consistent with a typical range of Z* in the North Pacific (0.8–1.0 km)^18^ than before (0.4–0.6 km) or for CW. Low Z* values denote the importance of upwelling relative to diffusion/mixing in determining the curvature of *θ* profiles.

More importantly, *J*/*w* values obtained from the *DO* profiles (for given Z* values) significantly varied in time and among water masses (Fig. 2e). In the 1990s, highly positive *J*/*w* values (more than +25 μmol kg^−1^ km^−1^) with an increasing trend were found for CW, and negative *J*/*w* values (less than −10 μmol kg^−1^ km^−1^) with a decreasing trend were found for DW, indicating increased (decreased) convective supply of DO to CW (DW). This implies a shift of the ventilation system from DW (particularly below DOM and BW) to CW above DOM in the 1990s, as previously suggested^1,2,10^. Note that the DOM had continuously deepened during the 1990s as compared to that towards the end of the 1970s (Figs 1b and 2f). In the 2000s, however, *J*/*w* for CW abruptly decreased and its sign changed from positive to negative with zero crossing in 2005, indicating reduced CW formation in the 2000s. On the other hand, *J*/*w* for DW clearly increased, approaching zero in 2016 (Fig. 2e) with shoaling of DOM and a less clear (smeared) structure in the DO profile (Figs 1b and 2f). Another shift in the ventilation system from CW back to DW below DOM (and BW) accounts for such *J*/*w* changes, and the consequent slowdown of the BW DO declining rates (Fig. 1c).

### Convective DO supply

The DO source term (*J*) is decomposed into the convective supply (*J*_*C*_) originating from the surface of northern ES and biological consumption (*J*_*B*_), i.e., *J* = *J*_*C*_ − *J*_*B*_. Then, the *J*_*C*_ can be expressed as follows^10^:

2 $${J}_{C}[{\rm{\mu }}\mathrm{mol}/\mathrm{kg}/\mathrm{year}]={C}_{0}\times Q/V$$

where *C*_0_ is surface water DO concentration in μmol kg^−1^, *Q* is volume transport from the surface to the deep-water in km^3^ year^−1^, and *V* is volume of the deep-water mass in km^3^. Then, the source term *J/w*

3 $$J/w={J}_{C}/w-{J}_{B}/w={C}_{0}/w\times Q/V-{J}_{B}/w$$

is estimated from time-varying *Q* and *V* obtained from the time rate of changes in CW and DW volumes assuming that *C*_0_*/w* and *J*_*B*_*/w* are constant in time. The magnitude and temporal structure of *J/w* values modelled using equation (3) for the period 1950–2060 (*Model J/w*) are consistent with those directly derived from observed vertical DO structures (vertical bars vs. symbols in Fig. 2e). Note that the *Q* and *V* time-series are calculated using linear and logarithmic fits of the CW–DW and DW–BW boundaries to observed vertical profiles to optimize the observational results. See the Methods section for details on calculation of *Model J/w* (vertical bars in Fig. 2e).

The two independent approaches (one from model equation (3) and the other directly from observations) commonly show the highly positive (negative) *J/w* in mid-1990 and abrupt decrease (increase) in mid-2000 for CW (DW). The consistency in absolute magnitude and temporal structure of *J/w*, in turn, indicates that our optimized fits with linear and logarithmic functions for selected periods to the observations provide reasonable estimates and projections within the RMS difference (~100 m) between fitted and observed depths of DSM and UBHL. The successful reconstruction of *J/w* with temporal changes in the deep-water boundary and volume suggests that *J/w* variations are primarily controlled by the rate of deep-water formation rather than biological consumption (appropriate assumption of time-varying *J*_*C*_ and constant *J*_*B*_). Thus, the slowdown of CW formation and re-initiation of ventilation into BW (and DW below DOM) account for the observed changes in the deep-water structure.

### Surface atmospheric and oceanic conditions

The CW–DW (DW–BW) boundary fits optimized with the 1-D model (red solid lines in Fig. 1b) and the DO source term modelled using equation (3) (*Model J/w*, vertical bars in Fig. 2e) were extended to 2060 for CW (DW), which provides a new projection of ventilation. The new projection suggests continuous volume reduction of deep-water masses but with changed rates for different waters. Rate of CW (DW and BW) reduction increase (decrease) because of the decreasing (increasing) CW (DW and BW) formation. BW is known to be formed by the slope convection of highly dense surface water along the continental slope in the northern coast, e.g., off Peter the Great Bay^3,22^ as observed during winter in 2000–2001^23–25^, whereas CW is considered to be formed by the open-ocean convection^3^ of surface water with relatively lower density. Thus, more BW formation implies that winter sea surface density (SSD) in the northern ES (a purple box in Fig. 1a) became higher in the recent decades, caused by factors such as more severe cooling, more brine rejection, less freshwater, and less heat fluxes. A previous box model study suggested that increased salt flux by the transport of the Tsushima Warm Current (TWC) enhanced SSD over northern ES from the 1990s to 2000s^15^, whereas this paper discusses changes in SSD by air-sea fluxes and the effect of brine rejection in the last decades, which can reasonably explain the observed features.

**Figure 3:**
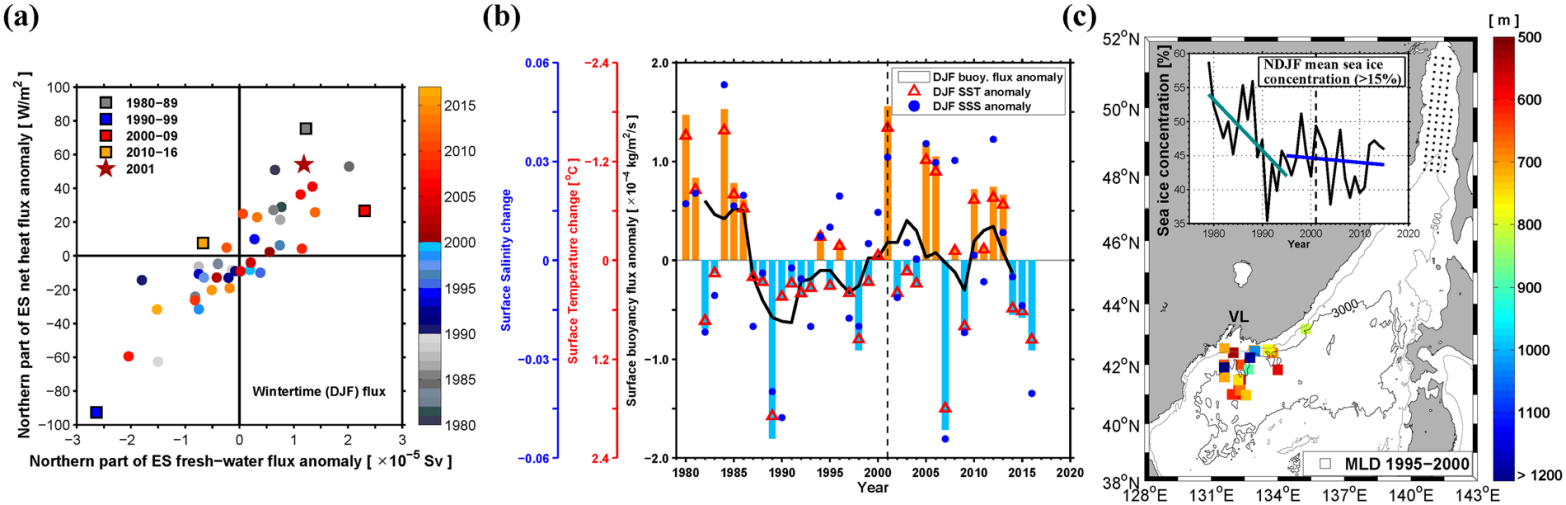
Winter atmospheric and oceanic conditions associated with deep-water formations in the northern East Sea. (**a**) Winter (December to February) fresh water flux anomaly (evaporation minus precipitation; positive when ocean loses freshwater) vs. heat flux anomaly (positive when ocean loses heat) during 1980–2016. The integrated anomalies of fresh water and heat fluxes for 10-year-segments starting from 1980 are shown with coloured squares (1980s, 1990s, 2000s, and 2010s are marked with grey, blue, red, and orange colours). The red star denotes the case of 2000–2001 winter when the slope convection was observed with sufficiently high sea surface density (SSD). (**b**) Changes in sea surface salinity (SSS, blue circle) and sea surface temperature (SST, red triangle), both averaged over the area marked with the purple box in Fig. 1a, regressed from the flux anomalies (see Methods). The surface buoyancy flux anomaly into (out of) the area is represented by vertical sky blue (orange) bars with the 5-year running mean (thick black line). The y-axes are for the changes of SSS (blue), SST (upside down, red), and surface buoyancy flux (black). (**c**) Locations where the mixed layer depth (MLD) deeper than 500 m was observed for the period 1995–2000, with a colour scale shown in the right. The top-left inset shows time series of sea ice concentration [%] (cases exceeding 15% only) averaged over the Tartarsky Strait region (marked with black dots) from November to February between 1979 and 2015 (black line), and its linear trends from 1979 to 1995 (green line) and from 1995 to 2015 (blue line). VL denotes Vladivostok, Russia. Figures were plotted using MATLAB R2016a (http://www.mathworks.com).

A positive linear relationship (correlation coefficient: 0.86) exists between anomalies of net heat and fresh water fluxes (heat flux is positive when ocean loses heat; fresh water flux is positive when evaporation exceeds precipitation) at the sea surface, which reinforced to control SSD during 1980–2016 (Fig. 3a). Positive net heat flux (cooling) and positive fresh water flux (salinization), which increase SSD, were more common either before 1990 or after 2000 than during the 1990s (Fig. 3a). Time-integrated fluxes show such contrasting conditions between the 1990s and 2000s (blue vs. red squares in Fig. 3a). During the winter between 2000 and 2001, when the deep slope convection was observed^22–25^, both heat and fresh water flux anomalies were strongly positive (red star in Fig. 3a). Sea surface temperature (SST) and sea surface salinity (SSS) averaged over the same area estimated by the winter surface flux anomalies (see Methods) clearly demonstrate cold and saline (high SSD) surface conditions in the 2000s and before 1990 compared to the 1990s (red triangles and blue circles in Fig. 3b). Surface buoyancy flux anomalies into the northern ES (bars in Fig. 3b) were quantified by combining the net heat and freshwater fluxes following the method previously suggested^26^. These anomalies showed more positive values (increasing SSD) in the last decades than the 1990s and the sign of its 5-year running mean time series shifted from negative to positive in the late 1990s and early 2000s (thick black line in Fig. 3b).

Because the SSD is more affected by the time-integrated freshwater flux (61%) than heat flux (39%) in the 2000s, contrasting other periods (red square in Fig. 3a), the increased SSS primarily (and decreased SST secondly) enhance slope convections in northern ES. The positive SSS anomaly averaged over the 2000s is two times stronger than the previously reported increase in horizontal salt flux due to the TWC effect^15^. Furthermore, changes in brine rejection with sea ice formation in the northern ES could also contribute to the increased SSS, and thus SSD since the late 1990s. Winter sea ice concentration in the Tartarsky Strait (area shown with black dots in Fig. 3c), which had a significant decreasing trend (−0.75 ± 0.49% year^−1^) before 1995 (green line in Fig. 3c), showed no significant decrease since the late 1990s. The sea ice concentration in the Tartarsky Strait was co-varied with that in front of the Vladivostok due to strong connection between the two regions via the Liman Current along the Primorye coast such that its variation is associated with deep and bottom water formations off Vladivostok. Thus, we believe the high SSD condition is caused primarily by increased SSS via enhanced air-sea freshwater flux (more evaporation) in northern ES, more brine rejection in the Tartarsky Strait, and/or more horizontal salt flux through the TWC^15^, and then secondarily by decreased SST via severe cooling in northern ES. All these factors are responsible for more active BW formation through the slope convection in the recent decades.

In contrast to the enhanced slope convection, open-ocean convection forming CW, represented by thick well-mixed layers^3^, decreased with reduced mixed layer depth (MLD). MLD was found to be deeper than 500 m, which is considered to be suitable for CW formation^3^, in 37 (among 1545 in total) *θ* profiles during 1995–2000 in northwestern JB, and the maximum MLD reached deeper than 1200 m (coloured symbols in Fig. 3c). However, such a deep (>500 m) MLD was found in only 2 (665 in total) *θ* profiles during 2000–2004 (after the winter of 1999–2000 when the latest open-ocean convection was observed^3^).

In short, surface atmospheric and oceanic conditions show significant decadal changes, which may affect the changes in water formation rates and long-term trends of deepening water boundaries (Figs 1b, 2e, 3b and c). However, the long-term trends of decreasing BW DO and deepening water boundaries have not been reversed despite the decadal variations (Fig. 1b and c), suggesting only slowdown of the long-term deepening (not shallowing) retained over multi-decades.

## Discussion and Conclusion

In this study, we analysed previous observations of a slowdown of long-term deepening in water boundaries associated with changes of water formation rates in the ES. Our findings consistently support the three-layered structure of deep-water masses in the ES without the disappearance of BW at least till 2040. The slowdown of the long-term deepening in water boundaries is supported by structural changes observed in the deep-water masses over past decades and results of the 1-D model applied to the central JB. According to previous studies^19,20,27,28^, the change in physical properties of deep-water masses in the eastern JB are comparable to that in the Yamato Basin (YB) located in the southeastern part of the ES although the absolute value of DO (*θ*) in the YB is lower (higher) than that in the eastern JB by 5 μmol kg^−1^ (0.015 °C)^20^. It is difficult to directly compare the rates observed in the two different basins because simultaneously observed data are not available. However, we infer that the findings in the central JB, quite representative for the entire ES, are not totally independent from the events taking place in the YB.

The results on vigorous changes in the ES ventilation system provide significant global implications. The markedly weakened ventilation from BW to CW in the mid-1990s is analogous to the ceasing of deep-water formation in the Southern Ocean over a course of a few hundred years projected under the global warming scenario. These results can be attributed to global warming, which restricts the formation of surface dense water in the deep-water formation areas. Thus, the past changes observed in the ES would provide a clue to future changes. The weakened ventilation is represented by changes in DO profiles from 1954 to 1979 to 1996 as more DO was supplied into CW with open-ocean convection and the CW volume was increased.

Moreover, the re-initiation of BW formation during the last decades, accompanied by changes in DO profiles from 1996 to 2005 to 2015 due to more DO supply into DW and BW and conditions favourable for the enhanced slope convection, yield the slowdown in declining DO in BW. This most recent change in the ventilation system is analogous to the enhanced deep ventilation, which was recently observed in the Labrador Sea where winter convective overturning to form the Labrador Sea Water has been observed since 1994 and reached to the deepest depth in 2015 due to a strong winter cooling event^29^. The stability of the Atlantic meridional overturning circulation is known to be quite sensitive to the hydrological cycle^30,31^ as well as the rate of CO_2_ increase^12,13,32^. Thus, the recent change in the ES ventilation system may provide another clue to future changes and support the need for long and continuous monitoring in the ES.

## Methods

### Hydrographic measurements

The hydrographic data used here were collected using Conductivity-Temperature-Depth (CTD) instruments via 19 research cruises between 1993 and 2016 (Table 1) conducted through CREAMS (Circulation Research of the East Asian Marginal Seas) and EAST (East Asian Seas Time-series)-I programs. The CTD stations cover most parts of the ES, particularly the deepest part (deeper than 3000 m) or Station C in central JB (centred around 134°E, 41.3°N; blue square in Fig. 1a), which was covered by nearly all cruises since the 1990s^2,4^. During the cruises, full-depth or shallow CTD casts were conducted at each station, and seawater samples were often collected at multiple discrete depths to analyse DO and other variables. Details on the hydrographic measurements are summarized in Table 1. In addition to these observations, vertical *θ* and DO profiles in the JB before 1994 were digitized using plots in previous works^2,10,18^ and were incorporated in the 1-D advection-diffusion model (Fig. 2).

**Table 1:** Details on CREAMS or EAST-I cruises conducted from 1993 to 2016. *T*, *S*, and DO denote water temperature, salinity, and dissolved oxygen, respectively.

CREAMS cruise date	Observation instrument	Number of stations	Observed variables	Pre- and Post- calibration	DO calibration	Remark	Reference
August 12–19, 1993	SBE911^a^	69	*T, S*			CTD casts down to 1000 dbar	4
July 10–21, 1994	SBE911	51	*T, S*				4
March 1–7, 1995	SBE911	35	*T, S*				4
July 24–Aug. 8, 1995	SBE911	45	*T, S*				4
February 17–24, 1996	SBE911	26	*T, S*				4
August 1–11, 1996	SBE911	35	*T, S*, DO				4
March 20–Apr. 8, 1997	SBE911	67	*T, S*, DO				4
February 22–Mar. 8, 1999	SBE911	43	*T, S*				4
June 14–August 13, 1999	NBMK^b^	203	*T, S*, DO		O		3, 38
February 2–March 17, 2000	NBMK	81	*T, S*				3, 5
April 16–20, 2001	NBMK	19	*T, S*				
April 7–26, 2002	SBE911p^c^	15	*T, S*				
October 15–27, 2005	SBE911p	43	*T, S*, DO	O			
May 10–20, 2007	SBE911p	23	*T, S*, DO				9
July 8–19, 2009	SBE911p	38	*T, S*, DO				
October 13–27, 2012	SBE911p	42	*T, S*, DO	O	O	1 Hz data sampling^d^	
April 15–29, 2014	SBE911p	28	*T, S*, DO	O	O		
April 6–May 3, 2015	SBE911p	36	*T, S*, DO	O	O		
April 5–15, 2016^e^	SBE911p	23	*T, S*, DO			South of 40.5°N, West of 132.5°E	

^a^Sea-Bird Electronics 9 CTD unit and Sea-Bird Electronics 11 deck unit.

^b^Neil Brown Instrument Systems MKIIIB CTD unit.

^c^Sea-Bird Electronics 9 plus CTD unit and Sea-Bird Electronics 11 plus V2 deck unit.

^d^Data sampling frequency of SBE911p is generally 24 Hz.

^e^Date of calibration of the DO sensor is February 5, 20.

### Ancillary data

To calculate net heat and fresh water fluxes at the sea surface, and changes in SST and SSS associated with the air-sea fluxes, monthly data (heat fluxes, air temperature, evaporation, and precipitation) from the Modern-Era Retrospective analysis for Research and Applications, Version 2 (MERRA2) for 1980–2016 with a horizontal resolution of 0.5° × 0.625°^33^, and monthly SST data from the optimally-interpolated (version 2) Advanced Very High Resolution Radiometer (AVHRR) for 1981–2011 with a resolution of 0.25° × 0.25°^34^ were used. A highly correlated linear relationship (*r* = 0.88) was found between the MERRA2 flux and the *S* observed at the uppermost depth during the 19 cruises in northern ES (purple box in Fig. 1a). Using the linear relationship at a lag of one month (flux leads), SSS change in winter (blue circles in Fig. 3b) was estimated from surface fresh water flux anomalies during 1980–2016. Similarly, SST change in winter (red triangles in Fig. 3b) was estimated from the net heat flux anomaly for the same period using the one-month-lag linear relationship between the flux and AVHRR SST for the area (*r* = −0.82, flux leads). The method above was used to determine changes in SSS and SST associated with surface air-sea fluxes only. The linear regression of net heat flux to SST explains 62% of variance in the AVHRR SST, indicating a significant role of air-sea heat exchange in determining SST.

Monthly sea ice concentration data with a grid size of 25 × 25 km from Nimbus-7 SMMR, DMSP SSM/I-SSMIS passive microwave data provided by the National Snow and Ice Data Center (NSIDC)^35^ were used to infer the SSS change relevant to brine rejection in the vicinity of the Tartarsky Strait. Winter sea ice concentration was calculated by averaging data from November to February for cases when the concentration exceeded 15% only^36^. The MLD was determined using hydrographic data collected in the northern ES via CTD casts, profiling floats, eXpendable Bathy Thermograph, and the World Ocean Database 2005 during 1995–2004. For this purpose, a threshold of temperature difference ${\rm{\Delta }}T=0.2\,^\circ {\rm{C}}$ and a reference depth of 10 m were set following previous works^37^. A total of 2210 MLDs in winter (November to March) was used in this study.

### Data processing

All CTD data were processed and carefully calibrated with raw data except, for data from 1999 June and 2000 February cruises, for which only preliminarily processed data are available. They were handled using standard data processing programmes, such as those of Sea-Bird Electronics (SBE), except for the AUTOSAL calibration and pre- and post-cruise sensor calibrations to correct the absolute bias additionally^38^. The absolute value of the CTD data were found to be potentially biased (within 0.001 °C in temperature and 0.002 in salinity) compared with the final processed data^3,5,38^. Most CTD data collected after 2002 were processed with both pre- and post-cruise calibrations, following typical SBE data processing sequences^39^. Although the effects of thermal mass^40–42^ on data quality is generally important, sensitivity tests with various combinations of input parameters indicate that the final processed *θ* and *S* below 500 m are nearly insensitive to the CellTM process, yielding an RMS difference of less than the sensor accuracy (0.001 °C in temperature and 0.001 in salinity).

For cases where post-cruise calibration was unavailable, sensor drift calibration and pre-cruise calibration were applied to correct absolute values of *θ* and *S*. The DO sensor data were calibrated using DO titrated from bottle samples by the Winkler method for cruises conducted in 1999, 2012, 2014, and 2015. For cases without bottle samples, DO profiles were calibrated by a drifting rate of DO at 3000 dbar assuming that BW DO linearly decreased between the periods^19^.

### Data analysis

The DSM is defined as a depth where salinity reaches its local minimum in the range of 600–3000 m, as considered previously^4^. The DSM in 2012 was much deeper than that in other years, which is partly because CTD data were not obtained at a sufficiently high sampling rate during that particular cruise (Table 1). The UBHL as a DW–BW boundary was estimated at a vertical *θ* gradient of less than 0.001 °C (temperature accuracy) per 100-m range. The UBHL was also estimated from calibrated sensor DO profiles following a method previously used^8^, which showed no marked difference from the UBHL derived using *θ* profiles (Fig. 1b).

**Figure 4:**
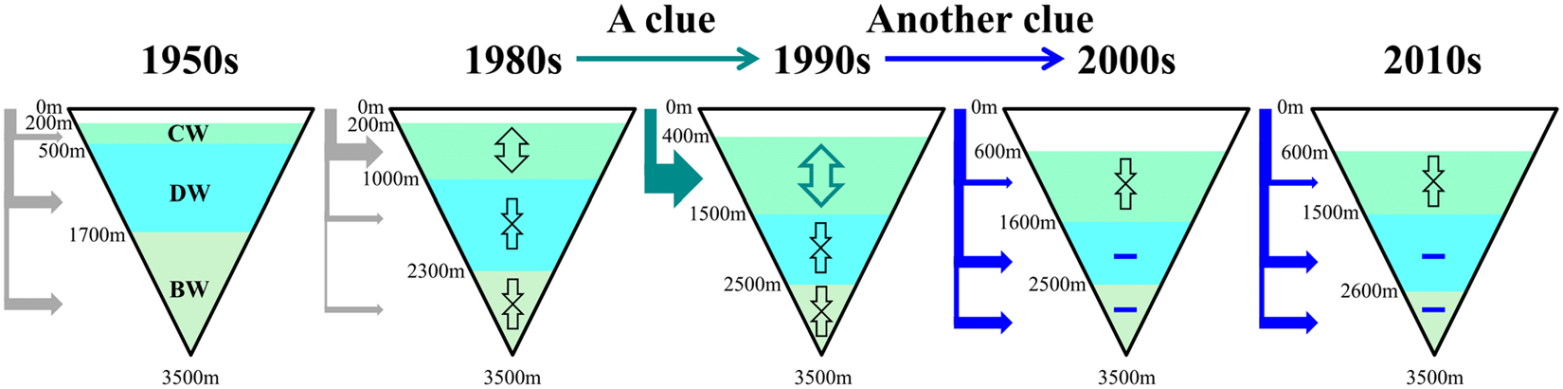
Schematics of changes in the ES ventilation since the 1950s. Vertical water boundaries are shown for representing periods (1950s, 1980s, 1990s, 2000s, and 2010s, with significant changes in 1990s and 2000s) following the volumes of deep-water masses (CW; Central Water, DW; Deep Water, and BW; Bottom Water). Here, the CW–DW boundary is based on 0.13–0.15 °C isotherm depth for the 1950s and 80s but DSM for 1990s, 2000s, and 2010s. Convective supplies of water volume (e.g., transport) and DO (e.g., flux) from the surface are marked with thick filled arrows where the formation of CW (DW and BW) via open-ocean convection (slope convection) is highlighted. Significant increasing and decreasing water volumes are shown with vertical arrows (increasing; one arrow heading both sides, decreasing: two arrows heading opposite directions). Recent slowdowns of reduction in DW and BW are marked by blue hyphens in 2000s and 2010s. Figures were generated using Adobe Illustrator CC 2015 (http://www.adobe.com/illustrator).

The upper boundary of CW, defined as an isotherm depth of 0.6 °C^4^ (plus symbols in Fig. 1b) was fitted to a second order polynomial for the period from 1994 to 2005 and a linear line for 2005–2060. Changes in water volume were then estimated using the fits to the boundary depth (red line in Fig. 1b) and the linear relationship between depth and area in the ES^43^ (cone shapes in Fig. 4). *J*_*B*_ for CW (DW) was estimated to be 1.73 (0.75) μmol kg^−1^ year^−1^ by the minimum complexity water mass model with chemical tracer data observed in the ES^44^. *C*_0_ was set to 320 μmol kg^−1^, as used previously^10^, yielding *J*_*B*_*/w* of 7.3 (1.4) μmol kg^−1^ km^−1^ for CW (DW) with the constant *C*_0_*/w* of 1350.2 (616.6) μmol kg^−1^km^−1^ year.
